# Supplementary figures and images for: Day and night camera trap videos are effective for identifying individual wild Asian elephants
Source: PeerJ. 2023 Mar 28;11:e15130. doi: 10.7717/peerj.15130 (PMC10064994; doi:10.7717/peerj.15130)

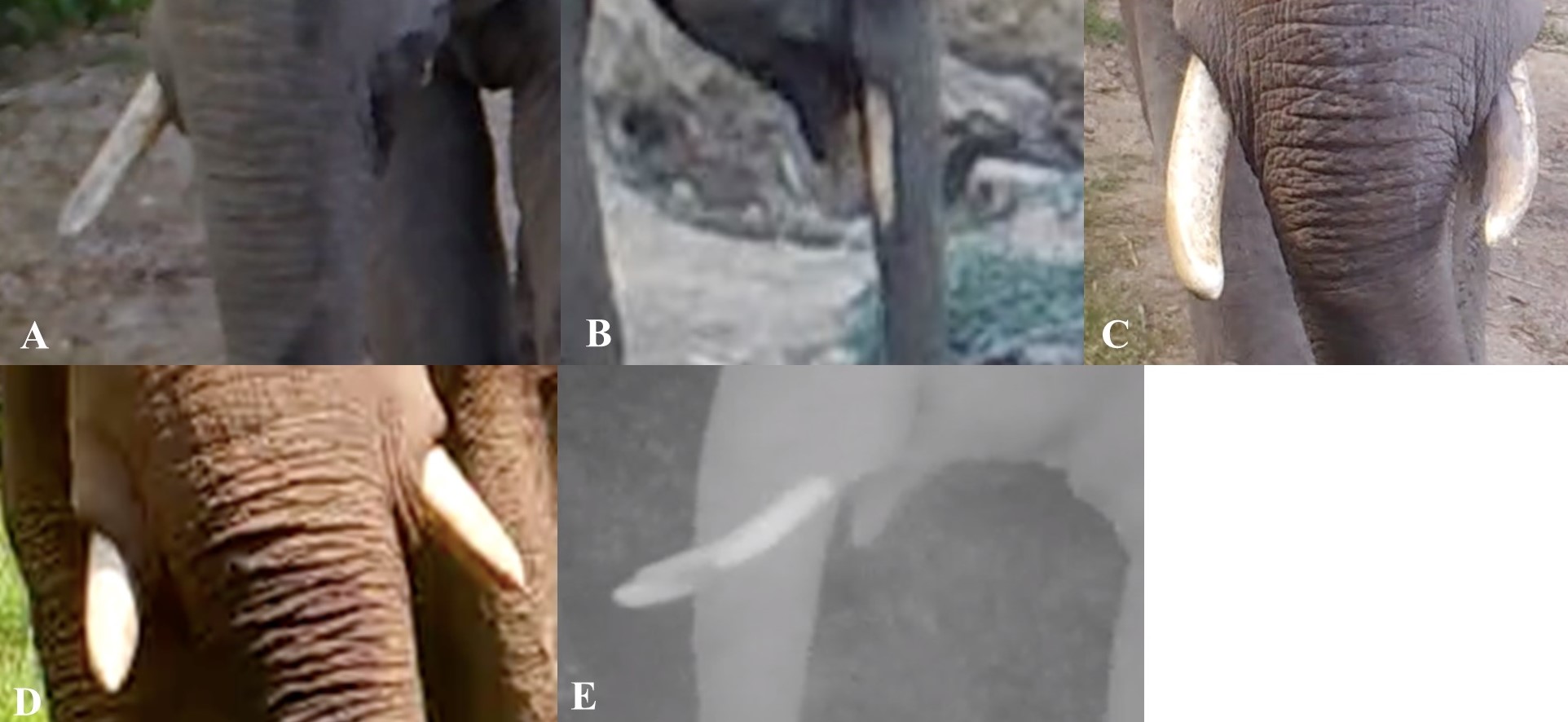

Supplement: Supplemental Information 1 — Table S4 provides detailed definitions/descriptions that correspond with each of the labeled images (Figs. S1A–S1E). The quality of the images is reduced due to their capture from video. [file peerj-11-15130-s001.jpg]

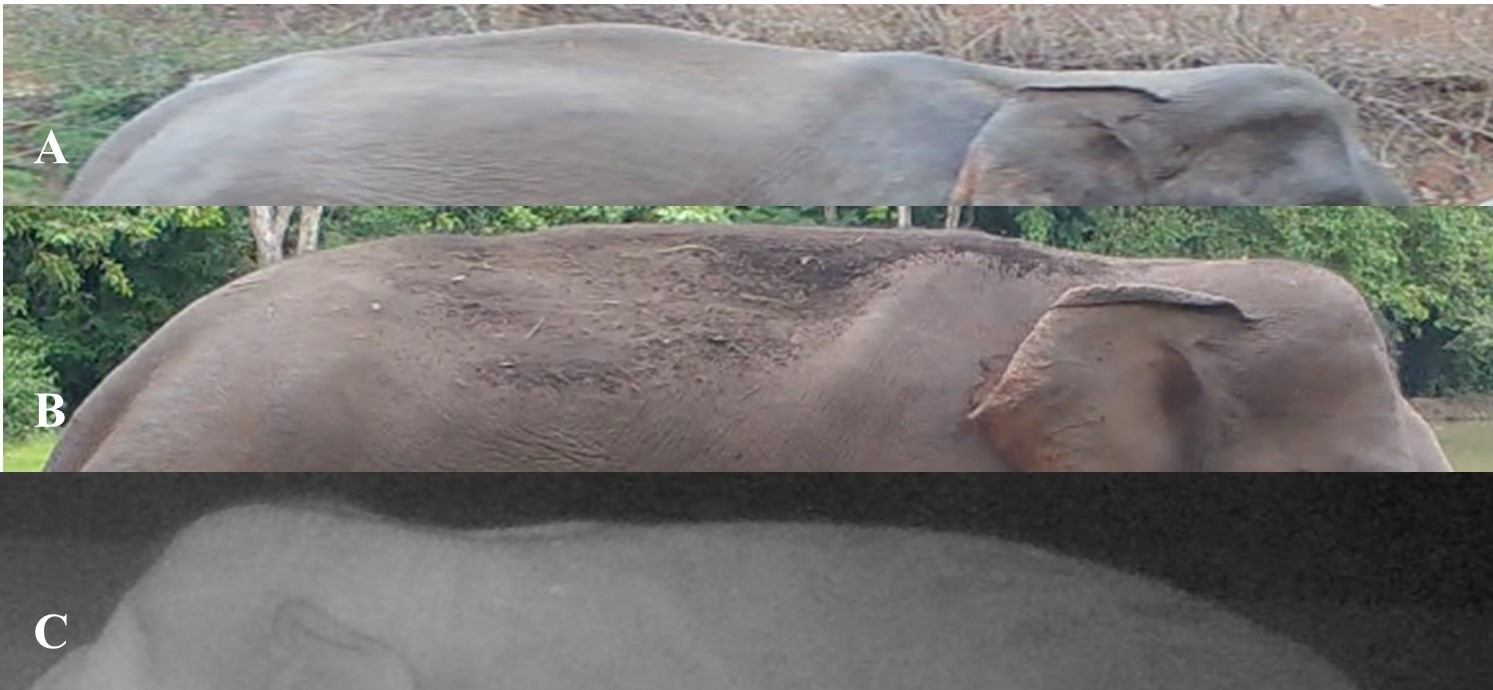

Supplement: Supplemental Information 2 — See Table S5 for descriptions of the back characteristics corresponding to the labeled images in the figure (Figs. S2A–S2C). [file peerj-11-15130-s002.jpg]

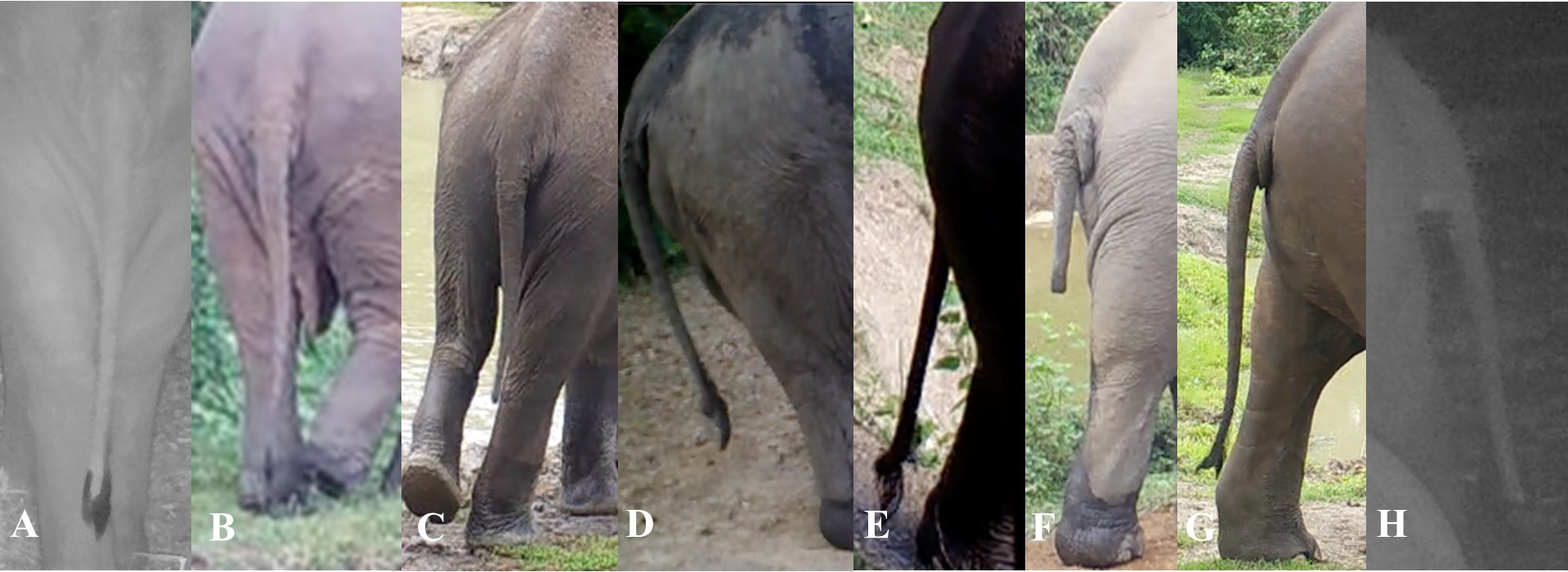

Supplement: Supplemental Information 3 — See Table S6 for descriptions of tail characteristics corresponding to the labeled images in the figure (Figs. S3A–S3H). [file peerj-11-15130-s003.jpg]

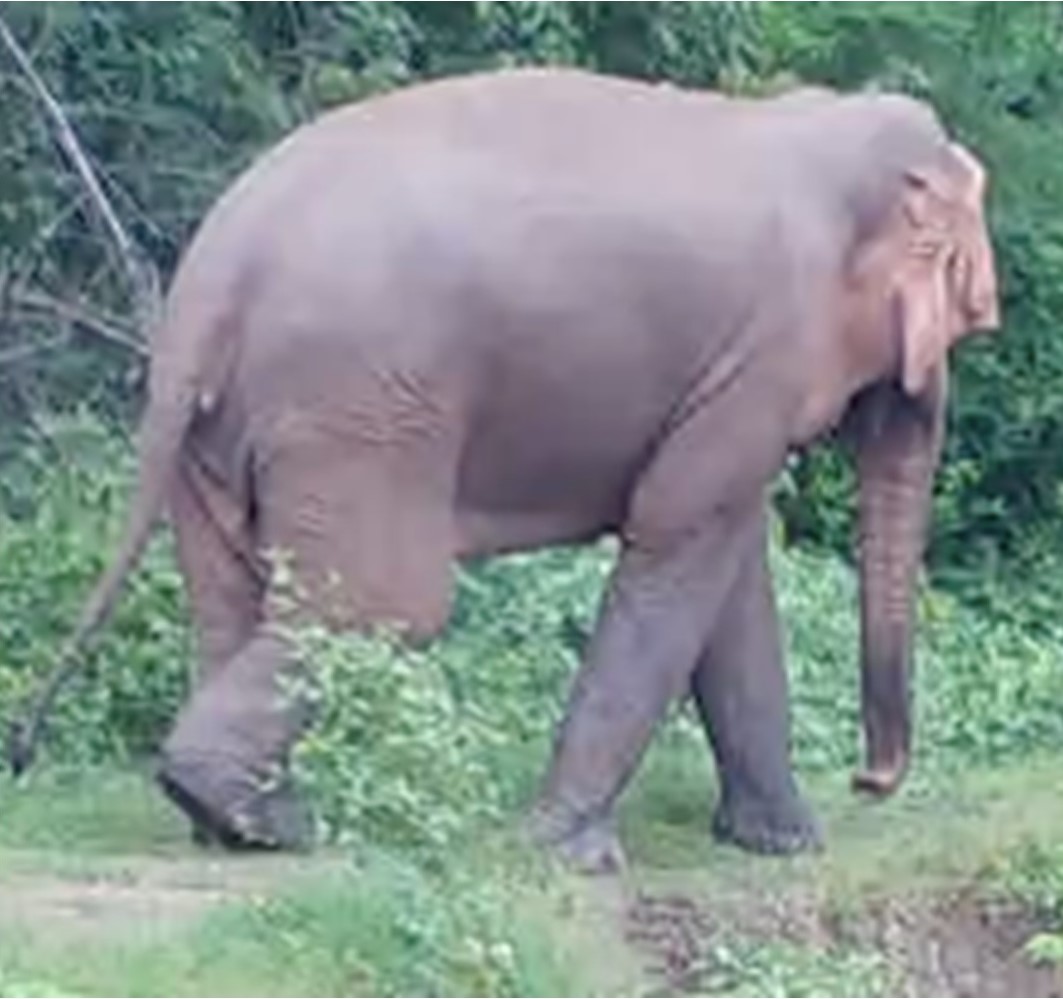

Supplement: Supplemental Information 4 — Note the different shades of pink on the elephant’s ear and underside of the trunk. [file peerj-11-15130-s004.jpg]
