# Supplementary material for: Day and night camera trap videos are effective for identifying individual wild Asian elephants
Source: PeerJ. 2023 Mar 28;11:e15130. doi: 10.7717/peerj.15130 (PMC10064994; doi:10.7717/peerj.15130)
Supplement: Supplemental Information 5 [file peerj-11-15130-s005.pdf]

Supplemental Material

Day and night camera trap videos are effective for identifying individual wild Asian elephants

Sasha Montero-De La Torre[1], Sarah L. Jacobson[1,2], Martin Chodorow[1,2], Marnoch Yindee[3,*], Joshua M. Plotnik[1,2,#]

[1] Department of Psychology, Hunter College, City University of New York, New York, NY

[2] Department of Psychology, The Graduate Center, City University of New York, New York, NY

[3] Akkhraratchakumari Veterinary College and One Health Research Centre, Walailak University, Nakhon Si Thammarat, Thailand

Correspondence:
There are Two Corresponding Authors (*, #)
*drfungy2000@yahoo.com; #Joshua.Plotnik@gmail.com

Table S1:
24 characteristics and the traits for identifying individuals

Characteristics adapted from Goswami et al. (2007); de Silva et al. (2013); Vidya et al. (2014). The combination column indicates that there could be two different traits on the right or left (R/L) side of the elephant (similar to Vidya et al., 2014). N/A was used if poor picture quality did not allow the trait to be determined or if only one side of the elephant was visible in videos.

Characteristic	Trait States	Combination
**Sex**	Male	
	Female	
	N/A	
**Age class**	A	
	B	
	C	
	D	
	N/A	
**Body condition**	0	
	1	
	2	
	N/A	
**Presence of tusks/tushes**	None	
	Both	
	R only	
	L only	
	N/A	
**Tusk Symmetry**	Even	
	Uneven	
	N/A	
**Tusk arrangement**	Parallel	
	Convergent	
	Splayed	
	N/A	
**Tusk angle**	Straight ahead	R/L
	Intermediate	
	Pointed down	
	N/A	
**Ear top fold**	None	R/L
	Forward slightly	
	Forward rolling fold	
	Forward flat fold	
	Backward	
	N/A	
**Ear side fold**	Forward	R/L
	Backward	
	N/A	
**Ear lobe shape**	L-angular	R/L

	V-acute<br>U-rounded<br>N/A	
**Ear tears**	None<br>At side fold<br>Before side fold<br>After side fold<br>On top fold<br>N/A	R/L
**Ear holes**	None<br>At side fold<br>Before side fold<br>After side fold<br>On top fold<br>N/A	R/L
**Ear depigmentation**	Present- Slight<br>Present- Prominent<br>None<br>N/A	R/L
**Tail length**	Stump (above abdomen)<br>Below genitals, above knee<br>Below knee, above ankle<br>At knee<br>At ankle<br>N/A	
**Brush type**	No hair<br>Short anterior<br>Short posterior<br>Short both<br>Short anterior normal posterior<br>Normal anterior short posterior<br>Normal anterior<br>Normal posterior<br>Normal both<br>N/A	
**Back shape**	Flat<br>Concave<br>Humped<br>N/A	
**Depigmentation on body**	Present on trunk<br>Present on body<br>Both<br>None<br>N/A	

Table S2:
Age classes and the trait state definitions

**Age**	**Trait State Definitions**
**Age Class**	*Adults (A):* around 15 years of age or older and, if female, had enlarged breasts or calves present
	Subadults (B): typically, 50-100% the size of adults or the same height without enlarged breasts and were approximately seven to fourteen years of age.
	Calves (C): identified if they could fit under the adults' belly or were within 50% of an adult's height and were approximately one to six years of age
	Infants (D): typically, less than a year in age and fit under the belly of an adult
	N/A: where age was not able to be determined due to quality of video or if genitalia was not visible

Table S3:
Body condition characteristic and trait state definitions

**Body Condition**	**Trait State Definitions**
**Body Condition**	*0*: the ribs, shoulder and pelvic girdles were prominent.
	1: ribs were not visible, shoulder and pelvic girdles were visible. Backbone was visible and pronounced
	2: shoulders and pelvic girdles were not visible, neck rolls, and plump. Backbone was not prominent.

Table S4:
Tusk characteristics and trait state definitions

Tusks/Tushes	Trait State Definitions	Examples
**Presence of Tusks**	*Both*: when an elephant had both of their tusks or tushes	Figures S1B-D
	Right only: when an elephant only had a right tusk or tush	Figure S1A
	Left only: when an elephant only had a left tusk or tush	
	None: when there were no tusks or tushes present for males or tushes present for females. If *none* was provided, the other tusk characteristics did not apply	
**Tusk Symmetry**	*Even*: when tusks were growing at an even rate	Figure S1D
	Uneven: when tusks were growing at an uneven rate or when one was broken (one tusk may be longer or shorter than the other)	Figure S1A, S1C
**Tusk Arrangement**	*Parallel*: tusks growing at the same angle, straight out, and pointing forward.	
	Splayed: tusks pointed outward (not parallel) from each other	Figure S1D
	Convergent: tusks growing out but inward, potentially resulting in tusks crossing over each other	Figure S1C
**Tusk Angle**	*Straight ahead*: tusks growing out parallel to a horizontal plane	Figure S1C, S1E
	Intermediate: tusks directed diagonally and not straight (parallel) or down (perpendicular)	
	Pointed down: tusks growing downward, perpendicular to the horizontal plane	Figure S1B

Table S5:
Back shape characteristics and trait state definitions

Back Characteristics	Trait State Definitions	Examples
**Back Shape**	*Flat:* where the majority of the back was more or less a straight line	Figure S2B, S2C
	Concave: where the back dipped in the center	
	Humped: where the back was elevated, primarily in the middle, but humps can occur throughout the back as well	Figure S2A

Table S6:
Tail characteristics and trait state definitions

Tail Characteristics	Trait State Definitions	Examples
**Tail Length**	*Stump (above abdomen):* a short and stubby tail that ended above the abdomen	Figure S3F
	Below genitals: above knee: a tail that extended between the genital area and above the knee	Figure S3D
	Below knee, above ankle: a tail that extended anywhere between the knee and the ankle	Figure S3A, S3G
	At knee: a tail that extended around the back of the knee	Figure S3C
	At ankle: a tail that extended to the ankle (before the leg becomes wider, forming the foot pad)	Figure S3B
**Brush Type**	*No hair:* there was no visible hair on the end of the tail	Figure S3C, S3F
	Short anterior: hair stubble, not long enough to naturally curve, on the side closest to the body	
	Short posterior: hair stubble, not long enough to naturally curve, on the side farthest from the body	
	Short both: hair stubble on both sides of the tail	
	Short anterior, normal posterior: hair stubble on the side closest to the body and normal hair length on the side farthest from the body	
	Normal anterior, short posterior: normal hair length on the side closest to the body and short/stubble hair on the side farthest from the body	
	Normal anterior: tail hair that is long enough to form its natural curve on the side closest to the body	Figure S3D, S3E, S3H
	Normal posterior: tail hair that is long enough to form its natural curve on the side farthest from the body	
	Normal both: tail hair that is long enough to form its natural curve on both sides of the tail	Figure S3A, S3B, S3G

Table S7:
Body depigmentation and trait state definitions

Depigmentation on Body	Trait State Definitions
**Depigmentation on body**	*Present on Trunk:* depigmentation was viewed on the trunk of the elephant
	Present on Body: depigmentation was viewed on the body of the elephant
	Both: depigmentation was viewed on both the body and trunk
	None: depigmentation was not found

Table S8:
Interrater reliability results of elephant characteristic categorization

Reliability was calculated for a subset of 30 videos of different individuals (19 daytime, 11 night); two data points from each video were included in the calculation for characteristics that were recorded on both the left and right side of an individual's body. The interpretation of level of agreement is based on benchmarks suggested by Landis & Koch (1977).

Characteristic	Cohen's kappa	Level of agreement
Presence of tusks	1	Excellent
Sex	0.81	Excellent
Tusk symmetry	0.74	Good
Tail brush	0.67	Good
Tusk angle	0.65	Good
Tusk arrangement	0.65	Good
Side fold	0.59	Moderate
Ear hole	0.58	Moderate
Ear lobe	0.54	Moderate
Tail length	0.53	Moderate
Top fold	0.47	Moderate
Ear tear	0.38	Fair
Ear depigmentation	0.36	Fair
Body condition	0.23	Fair
Back shape	0.15	Poor
Body depigmentation	0.13	Poor

Table S9:
Elephant count and calculation results for p_{max}^2 for all adult elephants (n=72), including most to least common characteristic and trait state option, excluding back shape and depigmentation on body

Ranked Characteristics	Majority Trait State	Number of elephants with trait	Proportion	Number of elephants with combination	p_{max}	p_{max}^2
**Presence of tusks/tushes**	None	63	0.875	63		
**L ear hole**	None	61	0.847	54	0.857	0.735
**Tail length**	Below knee, above ankle	58	0.8556	43	0.697	0.485
**L ear side fold**	Backward	56	0.778	35	0.698	0.487
**R ear hole**	None	56	0.778	28	0.557	0.311
**R ear side fold**	Backward	55	0.764	27	0.673	0.453
**R ear depigmentation**	Present-Prominent	53	0.736	21	0.434	0.188
**Sex**	Male	52	0.722	14	0.449	0.201
**Body condition**	1	49	0.681	11	0.341	0.116
**L ear depigmentation**	Present-Prominent	48	0.66	11	0.449	0.201
**R ear lobe shape**	V-acute	47	0.653	6	0.186	0.035
**L ear lobe shape**	V-acute	46	0.639	6	0.449	0.201
**Brush type**	Normal both	42	0.583	5	0.155	0.24
**R ear tear**	At side fold	37	0.514	2	0.179	0.032
**L ear top fold**	Forward rolling fold	31	0.431	2	0.155	0.24
**R ear top fold**	Forward rolling fold	31	0.431	2	0.179	0.032
**L ear tear**	At side fold	29	0.403	1	0.077	0.006
**Tusk symmetry**	Uneven	5	0.069	0	0	0
**R tusk angle**	Straight ahead	5	0.069	-	-	-
**L tusk angle**	Straight ahead	4	0.054	-	-	-
**Tusk arrangement**	N/A	3	0.042	-	-	-

Table S10:
Elephant count and calculation results for p_{max}^2 for adult male elephants (n=52), including most to least common characteristic and trait state option

Ranked Characteristics	Majority Trait State	Number of elephants with trait	Proportion	Number of elephants with combination	p_{max}	p_{max}^2
**Back shape**	Humped	46	0.885	46		
**Presence of tusks**	None	43	0.827	37	0.804	0.647
**L ear hole**	None	42	0.808	30	0.717	0.514
**Tail length**	Below knee, above ankle	42	0.808	23	0.617	0.380
**Depigmentation on body**	Both	41	0.788	18	0.561	0.315
**L ear side fold**	Backward	40	0.769	16	0.548	0.300
**R ear depigmentation**	Present-Prominent	38	0.731	12	0.421	0.177
**R ear side fold**	Backward	37	0.712	12	0.548	0.300
**R ear hole**	None	37	0.712	10	0.351	0.123
**L ear depigmentation**	Present-Prominent	33	0.635	10	0.548	0.300
**Body condition**	1	31	0.596	8	0.281	0.079
**R ear lobe shape**	V-acute	31	0.596	5	0.343	0.117
**L ear lobe shape**	V-acute	29	0.558	5	0.281	0.079
**R ear tear**	At side fold	29	0.558	3	0.206	0.042
**Brush type**	Normal both	28	0.538	2	0.187	0.035
**R ear top fold**	Forward rolling fold	24	0.462	2	0.206	0.042
**L ear top fold**	Forward rolling fold	23	0.442	2	0.187	0.035
**L ear tear**	At side fold	23	0.442	1	0.103	0.011
**Tusk symmetry**	Uneven	5	0.096	0	0	0
**R tusk angle**	Straight ahead	5	0.096	-	-	-
**L tusk angle**	Straight ahead	4	0.077	-	-	-
**Tusk arrangement**	N/A	3	0.058	-	-	-

Table S11:
Elephant count and calculation results for p_{max}^2 for adult male elephants (n=52), including most to least common characteristic and trait state option, excluding back shape and depigmentation on body

Ranked Characteristics	Majority Trait State	Number of elephants with trait	Proportion	Number of elephants with combination	p_{max}	p_{max}^2
**Presence of tusks**	None	43	0.827	43		
**L ear hold**	None	42	0.808	35	0.814	0.663
**Tail length**	Below knee, above ankle	42	0.808	28	0.662	0.438
**L ear side fold**	Backward	40	0.769	24	0.698	0.487
**R ear depigmentation**	Present-Prominent	38	0.731	17	0.469	0.220
**R ear side fold**	Backward	37	0.712	16	0.657	0.431
**R ear hole**	None	37	0.712	14	0.410	0.168
**L ear depigmentation**	Present-Prominent	33	0.635	14	0.657	0.431
**Body condition**	1	31	0.596	11	0.322	0.104
**R ear lobe shape**	V-acute	31	0.596	6	0.358	0.128
**L ear lobe shape**	V-acute	29	0.558	6	0.322	0.104
**R ear tear**	At side fold	29	0.558	3	0.179	0.032
**Brush type**	Normal both	28	0.538	2	0.215	0.046
**R ear top fold**	Forward rolling fold	24	0.462	2	0.179	0.032
**L ear top fold**	Forward rolling fold	23	0.442	2	0.215	0.046
**L ear tear**	At side fold	23	0.442	1	0.090	0.008
**Tusk symmetry**	Uneven	5	0.096	0	0	0
**R tusk angle**	Straight ahead	5	0.096	-	-	-
**L tusk angle**	Straight ahead	4	0.077	-	-	-
**Tusk arrangement**	N/A	3	0.058	-	-	-

Table S12:
Elephant count and calculation results for p_{max}^2 for adult female elephants (n=20), including most to least most common characteristic and trait state option

Ranked Characteristics	Majority Trait State	Number of elephants with trait	Proportion	Number of elephants with combination	p_{max}	p_{max}^2
**Presence of tusks/tushes**	None	20	1.00	20		
**L ear hole**	None	19	0.95	19	0.950	0.903
**R ear hole**	None	19	0.95	18	0.947	0.898
**Body condition**	1	18	0.90	16	0.844	0.713
**R ear side fold**	Backward	18	0.90	15	0.888	0.789
**Back shape**	Humped	17	0.85	13	0.732	0.536
**L ear lobe shape**	V-acute	17	0.85	10	0.683	0.467
**L ear side fold**	Backward	16	0.80	9	0.659	0.434
**R ear lobe shape**	V-acute	16	0.80	8	0.683	0.467
**Tail length**	Below knee, above ankle	16	0.80	8	0.585	0.343
**L ear depigmentation**	Present-Prominent	15	0.75	5	0.427	0.182
**R ear depigmentation**	Present-Prominent	15	0.75	5	0.585	0.343
**Brush type**	Normal both	14	0.70	4	0.342	0.117
**L ear tear**	None	11	0.55	2	0.439	0.193
**Depigmentation on body**	Both	11	0.55	2	0.228	0.052
**R ear tear**	None	10	0.50	1	0.220	0.048
**L ear top fold**	Forward rolling fold	8	0.40	0	0	0
**R ear top fold**	Forward slightly	8	0.40	-	-	-

Table S13:
Elephant count and calculation results for p_{max}^2 for adult female elephants (n=20), including most to least most common characteristic and trait state option, excluding back shape and depigmentation on body

Ranked Characteristics	Majority Trait State	Number of elephants with trait	Proportion	Number of elephants with combination	p_{max}	p_{max}^2
**Presence of tusks/tushes**	None	20	1.00	20		
**L ear hole**	None	19	0.95	19	0.950	0.903
**R ear hole**	None	19	0.95	18	0.947	0.898
**Body condition**	1	18	0.90	16	0.844	0.713
**R ear side fold**	Backward	18	0.90	15	0.888	0.789
**L ear lobe shape**	V-acute	17	0.85	12	0.676	0.456
**L ear side fold**	Backward	16	0.80	10	0.740	0.548
**R ear lobe shape**	V-acute	16	0.80	10	0.676	0.456
**Tail length**	Below knee, above ankle	16	0.80	9	0.666	0.444
**L ear depigmentation**	Present-Prominent	15	0.75	6	0.450	0.203
**R ear depigmentation**	Present-Prominent	15	0.75	6	0.666	0.444
**Brush type**	Normal both	14	0.70	5	0.375	0.141
**L ear tear**	None	11	0.55	4	0.533	0.284
**R ear tear**	None	10	0.50	3	0.281	0.079
**L ear top fold**	Forward rolling fold	8	0.40	0	0	0
**R ear top fold**	Forward slightly	8	0.40	-	-	-

References Cited in Supplemental Tables

de Silva S, Webber CE, Weerathunga US, Pushpakumara TV, Weerakoon DK, Wittemyer G. 2013. Demographic variables for wild Asian elephants using longitudinal observations. *PLOS ONE* **8(12)**:e82788. DOI: 10.1371/journal.pone.0082788.

Goswami VR, Madhusudan MD, Karanth KU. 2007. Application of photographic capture-recapture modelling to estimate demographic parameters for male Asian elephants. *Animal Conservation* **10(3)**:391–399. DOI: 10.1111/j.1469-1795.2007.00124.x.

Landis JR, Koch GG. 1977. The measurement of observer agreement for categorical data. *Biometrics*, **33(1)**:159-174. DOI: 10.2307/2529310.

Vidya TNC, Prasad D, Ghosh A. 2014. Individual identification in Asian elephants. *Gajah* **40**:3–17.
